# Interaction of New-Developed TiO_2_-Based Photocatalytic Nanoparticles with Pathogenic Microorganisms and Human Dermal and Pulmonary Fibroblasts

**DOI:** 10.3390/ijms18020249

**Published:** 2017-01-25

**Authors:** Ionela Cristina Nica, Miruna Silvia Stan, Marcela Popa, Mariana Carmen Chifiriuc, Veronica Lazar, Gratiela G. Pircalabioru, Iuliana Dumitrescu, Madalina Ignat, Marcel Feder, Liviu Cristian Tanase, Ionel Mercioniu, Lucian Diamandescu, Anca Dinischiotu

**Affiliations:** 1 Department of Biochemistry and Molecular Biology, Faculty of Biology, University of Bucharest, 91-95 Splaiul Independentei, 050095 Bucharest, Romania; cristinai.nica@gmail.com (I.C.N.); miruna.stan@bio.unibuc.ro (M.S.S.); 2 Department of Botanic-Microbiology, Faculty of Biology, University of Bucharest, 1-3 Aleea Portocalelor, 60101 Bucharest, Romania; bmarcelica@yahoo.com (M.P.); veronica.lazar2009@gmail.com (V.L.); 3 Research Institute of the University of Bucharest—ICUB, University of Bucharest, 91-95 Splaiul Independentei, 050095 Bucharest, Romania; gratiela87@gmail.com; 4 National R&D Institute for Textiles and Leather Bucharest (INCDTP), 16 Lucretiu Patrascanu, 030508 Bucharest, Romania; iuliana.dumitrescu@certex.ro; 5 National Research and Development Institute for Textiles and Leather (INCDTP) Leather and Footwear Research Institute (ICPI), 93 Ion Minulescu, 030215 Bucharest, Romania; madalina.fleancu@yahoo.com; 6 National Institute of Materials Physics (NIMP), Atomistilor 405A, 077125 Bucharest-Magurele, Romania; mfeder@infim.ro (M.F.); liviu.tanase@infim.ro (L.C.T.); imercioniu@infim.ro (I.M.)

**Keywords:** photocatalyst, titania, antibacterial, skin, lung fibroblasts

## Abstract

TiO_2_-based photocatalysts were obtained during previous years in order to limit pollution and to ease human daily living conditions due to their special properties. However, obtaining biocompatible photocatalysts is still a key problem, and the mechanism of their toxicity recently received increased attention. Two types of TiO_2_ nanoparticles co-doped with 1% of iron and nitrogen (TiO_2_-1% Fe–N) atoms were synthesized in hydrothermal conditions at pH of 8.5 (HT1) and 5.5 (HT2), and their antimicrobial activity and cytotoxic effects exerted on human pulmonary and dermal fibroblasts were assessed. These particles exhibited significant microbicidal and anti-biofilm activity, suggesting their potential application for microbial decontamination of different environments. In addition, our results demonstrated the biocompatibility of TiO_2_-1% Fe–N nanoparticles at low doses on lung and dermal cells, which may initiate oxidative stress through dose accumulation. Although no significant changes were observed between the two tested photocatalysts, the biological response was cell type specific and time- and dose-dependent; the lung cells proved to be more sensitive to nanoparticle exposure. Taken together, these experimental data provide useful information for future photocatalytic applications in the industrial, food, pharmaceutical, and medical fields.

## 1. Introduction

Although the diversity of manufactured nanomaterials (MNMs) is very high, there is currently a continuous development of the technologies used to create new materials with improved properties [1,2]. These include titanium dioxide (TiO_2_), which is usually utilized in a large variety of consumer products, such as paints, varnishes, plastics [3,4], cosmetics, pharmaceuticals [5], and skin care products [6]. More recently, due to their unusual physicochemical properties, titanium dioxide nanoparticles (TiO_2_ NPs) have begun to be used as photocatalysts in air cleaning [7], wastewater management [8], hydrogen production [9], orthodontic composites [10,11], anticorrosion coatings for dental and bone implants, self-cleaning surfaces, and medical instruments [12].

On surfaces covered with a thin layer of TiO_2_, inactivation of microorganisms was observed as a result of advanced oxidation processes (AOP) initiated by ultraviolet radiation (UV) [13], which represents only 5% of terrestrial sunlight [14]. Hence, new nanomaterials with enhanced photocatalytic efficiency have been synthesized and tested. TiO_2_ nanoparticles were doped with metals (Fe) and non-metals (N) atoms to expand the photocatalytic performance in visible light [15].

However, the technological progress has brought a continuous need for checks in the safety of these nano-products for the environment and human health. What is known so far, from the nanotoxicological studies, is that the same properties which make MNMs both fascinating and useful are also reasons of serious concerns [16,17,18]. Recent investigations revealed that the lung represents the main target organ following workers’ exposure to NPs via inhalation [19], and the particles mainly deposit (75%–80%) in the alveolar region where they could interfere with, or within, cells [20]. Other possible methods of exposure include contact through the skin and ingestion, and the physico-chemical parameters of NPs, such as size and charge, seem to have an important impact on dermal penetration, and also on gastrointestinal absorption [21].

Different parameters can determine the cytotoxicity of nanomaterials, but the main concern is that they are redox active and due to their small size, some particles can penetrate the cell membranes and have a greater potential to generate oxidative stress [22]. Several studies, carried out recently both in vitro and in vivo, have suggested that the production of reactive oxygen species (ROS), especially after exposure to visible light or UV, is the main marker of cytotoxicity of TiO_2_ nanoparticles and the main responsible mechanism for the inflammatory lung effects induced by inhalation [23,24]. At the cellular level, ROS may be generated directly from the particles near, or within, the cell, or indirectly as a result of internalized particles on mitochondrial respiration, as well as due to the antioxidant species depletion [25]. After all, TiO_2_ nanoparticles can cause respiratory toxicity and epithelial inflammation, but compared to other nanoparticles these ones have a low or insignificant cytotoxicity [26].

It is well known the material properties depend strongly on the raw materials, synthesis method, time, temperature, pressure, etc. In wet methods (like hydrothermal processes) the pH of the solution can trigger the structure, morphology, and photocatalytic properties as well. Taking into account this aspect, in the present study, two types of TiO_2_ nanoparticles co-doped with 1% atoms of iron and nitrogen (TiO_2_-1% Fe–N) synthesized under hydrothermal conditions, using two different pH levels were considered. The structure and morphology of these NPs were investigated by X-ray diffraction (XRD), transmission electron microscopy (TEM), and X-ray photoelectron spectroscopy (XPS), and compared with those of reference material P25 (Degussa) and 1% Fe–N impregnated P25. The photocatalytic properties of these powders, on the degradation of methylene blue (MB), were comparatively revealed by a photocatalytic checker. Further, the new-developed TiO_2_-1% Fe–N particles were investigated in terms of antimicrobial efficiency and biological interaction with human cells. Cytotoxic effects were assessed in vitro on human dermal (CCD-1070Sk cell line) and pulmonary (MRC-5 cell line) fibroblasts by several parameters, including cell viability, antioxidant response and lipid peroxidation. Further, the antimicrobial activity was tested against Gram-positive fungal (*Candida albicans*) and bacterial (*Staphylococcus aureus*, *Enterococcus faecalis*) strains and Gram-negative (*Escherichia coli*, *Pseudomonas aeruginosa*) bacterial strains by quantitative methods according to international standards.

## 2. Results

### 2.1. Characterization of TiO_2_ Nanoparticles (NPs)

#### 2.1.1. X-ray Diffraction Results

**Figure 1:**
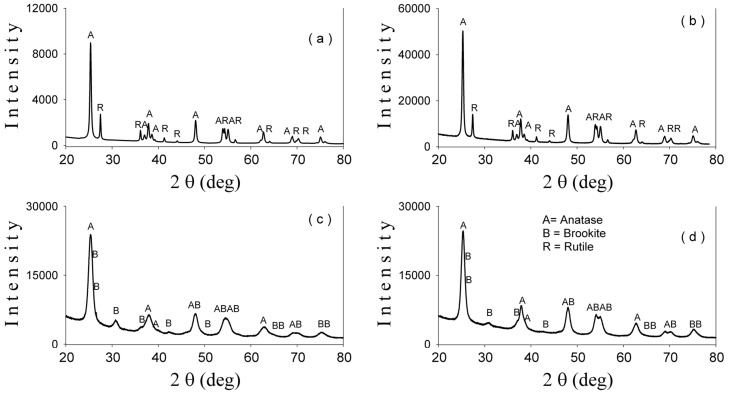
X-ray diffraction (XRD) patterns and phase assignment of the samples: P25 (**a**); P25-1% Fe–N (**b**); TiO_2_-1% Fe–N pH ~8.5 (**c**); and TiO_2_-1% Fe–N pH ~5.5 (**d**).

In Figure 1 the X-ray diffractograms of the analyzed particles are depicted. The corresponding Rietveld refinement results are presented in Table 1. The samples P25 and P25-1% Fe–N consist in two nanoscaled phases: a prevailing anatase phase (83%) accompanied by ~17% rutile phase. The crystallite size of anatase phase—close to 30 nm—is generally lower that of rutile one. In contradistinction, the samples TiO_2_-1% Fe–N pH ~5.5 and TiO_2_-1% Fe–N pH ~8.5 consist in nanoscaled anatase (~80%) and brookite (~20%) for the first sample, and anatase (~85%) and brookite (~15%) for the sample synthesized at pH ~8.5. The crystallite sizes of these two samples are much lower than of the P25 series (Table 1). It can be observed that the hydrothermal treatment of the samples leads to a drop in crystallite size.

The existence of iron in the prepared particles was firstly revealed within the Mössbauer spectroscopy. Only Fe^3+^ was identified in the hydrothermally-synthesized samples. A detailed analysis showing the site occupancy to Fe^3+^ in the samples was given in our previous work [27].

#### 2.1.2. X-ray Photoelectron Spectroscopy (XPS) Results

**Figure 2:**
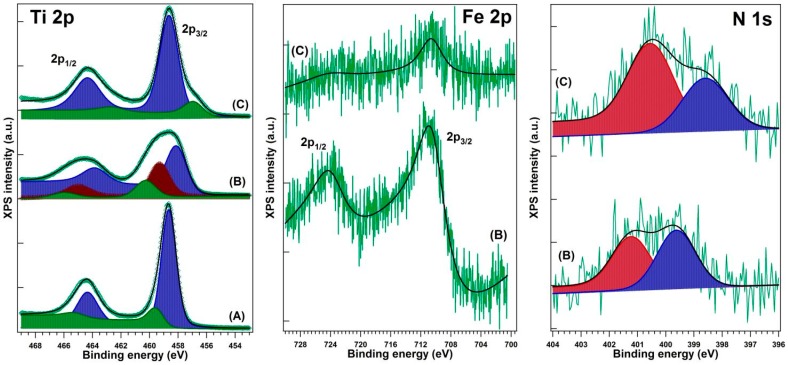
The X-ray photoelectron spectroscopy (XPS) spectra recorded on P25 TiO_2_ (**A**); P25-1% Fe–N (**B**); and TiO_2_-1% Fe–N (pH ~8.5) (**C**) samples.

The evidence for the presence of iron and nitrogen in the analyzed samples was given by XPS measurements. Representative XPS spectra of Ti 2p, Fe 2p, and N 1s are presented in Figure 2, while Table 2 contains the corresponding binding energies. All Ti 2p spectra exhibit a component at 458.08–458.88 eV, which is a pattern of Ti^4+^ state in TiO_2_ [28]. The 459.53 eV component in P25 and 459.30 eV in the P25-1% Fe–N sample can be associated with local defects associated with oxygen vacancies in the TiO_2_ structure. The 460.27 eV may correspond to a shake-up satellite, while the 456.88 eV peak indicates the presence of Ti^3+^ ions [28,29], probably through the formation of a small amount of TiFeO_3_-like compound, considering also the Fe^3+^ chemical state of iron. A more detailed analysis was previously presented [30].

#### 2.1.3. Morphological Characterization

**Figure 3:**
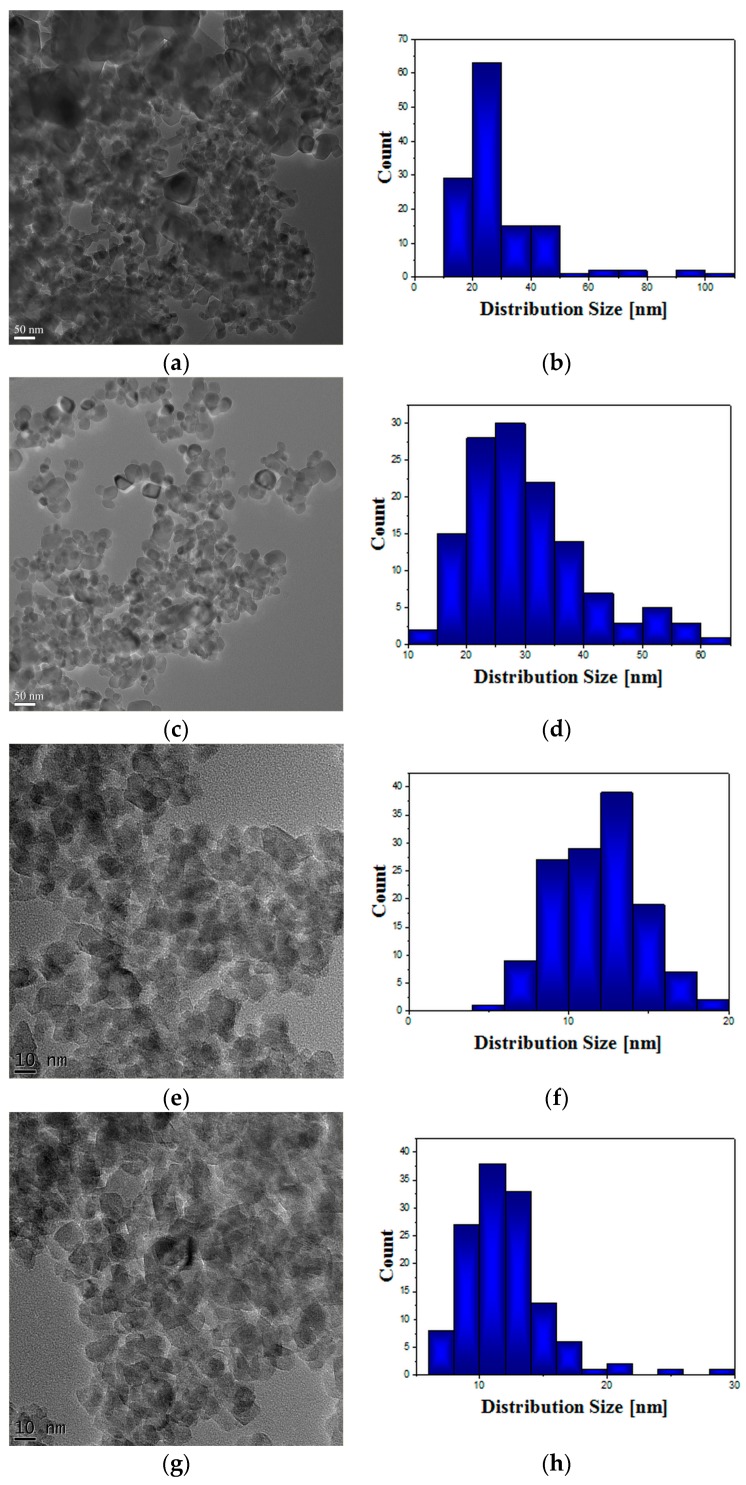
Transmission electron microscopy (TEM) images and particle distribution diagrams of the following samples: P25 (**a**,**b**); P25-1% Fe–N (**c**,**d**); TiO_2_-1% Fe–N (pH ~5.5) (**e**,**f**); and TiO_2_-1% Fe–N (pH ~8.5) (**g**,**h**).

Transmission electron microscopy (TEM) images (Figure 3) display the powder morphology of nanoscaled photocatalysts together with the corresponding particle distribution. More than 100 particles were considered for statistical measurements. The P25 particles have a polyhedral shape with round corners. A mean size of 29 ± 15 nm characterized the P25 sample while the mean particle size of P25-1% Fe–N was 30 ± 10 nm (Table 3). The sample TiO_2_-1% Fe–N (pH ~5.5) and TiO_2_-1% Fe–N (pH ~8.5) exhibited a mean size of 15 ± 2.8 nm and of 10 ± 4 nm, respectively, in rather good agreement with the XRD results. The hydrothermal sample obtained at pH ~8.5 showed a prevailing quadratic morphology, while a combined morphology (triangular, spherical, etc.) characterized the sample obtained at pH ~5.5.

**Figure 4:**
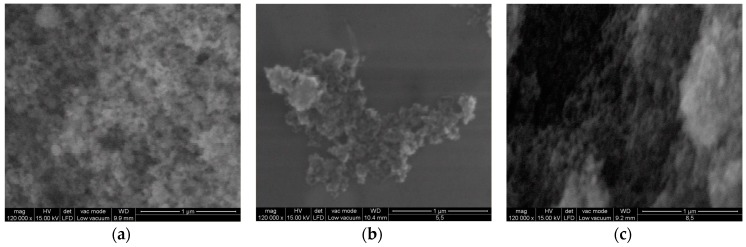
Scanning electron microscopy (SEM) images of P25 (**a**); TiO_2_-1% Fe–N, pH ~5.5 (**b**); and TiO_2_-1%-Fe–N, pH ~8.5 (**c**).

Figure 4 showed the surface morphology of TiO_2_-1% Fe–N at pH ~5.5 and TiO_2_-1% Fe–N at pH ~8.5 compared to that of P25 after scanning electron microscopy (SEM) analysis. Due to their small size (from 77.7 to 105.5 nm for P25, 300.4–511.3 nm for TiO_2_-1% Fe–N at pH ~5.5 and 79.9–112.3 nm for TiO_2_-1% Fe–N at pH ~8.5) and their high relative surface, the particles formed large agglomerates.

#### 2.1.4. UV-VIS Distribution

**Figure 5:**
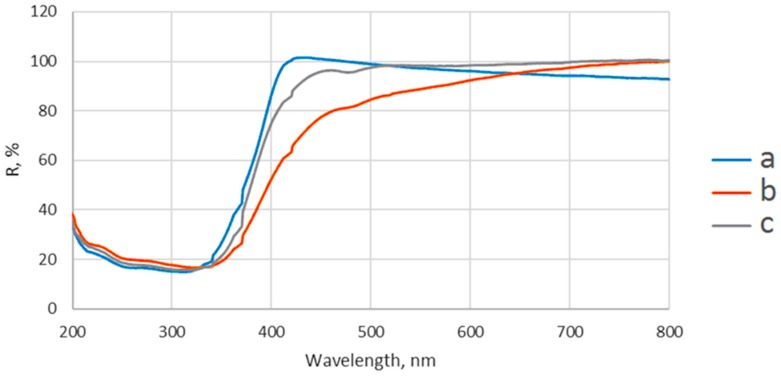
UV-VIS diffuse reflectance spectra of P25 (**a**); TiO_2_-1% Fe–N, pH ~5.5 (**b**); and TiO_2_-1% Fe–N, pH ~8.5 (**c**) samples.

The UV-VIS diffuse reflectance spectra of P25 and hydrothermally-synthesized samples are shown in Figure 5. It can be seen that the TiO_2_ particles exhibited a slight shift of the absorption edge from 347 nm for P25 to 350 nm for TiO_2_-1% Fe–N, pH ~8.5 and to 352 nm for TiO_2_-1% Fe–N, pH ~5.5. The high diffuse reflectance of P25 around 428 nm was attributed by other researchers to the luminescence effects generated by de-excited electrons under visible light [31]. The absorption shifting to visible region of N and Fe co-doped TiO_2_ particles reported previously by other studies [32,33] strongly depends on preparation and characterization methods.

#### 2.1.5. Photocatalytic Activity

**Figure 6:**
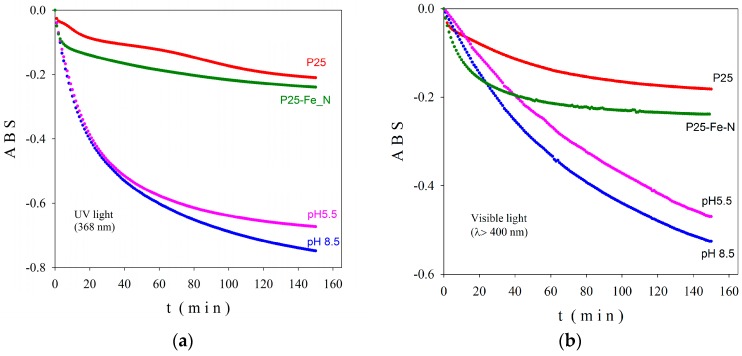
Absorbance (ABS) for the investigated samples in UV (**a**); and visible light (**b**).

The absorbance (ABS) measured by the photocatalytic checker on films, prepared as described in Materials and Methods Section, is presented in Figure 6 versus the irradiation time in UV (a) and visible light (b). ABS reflects the decomposition in time of methylene blue (MB) as a result of photocatalytic effect of the TiO_2_ based films; higher is the negative ABS value, better is the activity of the analyzed photocatalyst.

The TiO_2_-1% Fe–N, pH ~8.5 photocatalyst was able to decompose in 150 min nearly 80% of MB in UV light and over 50% in visible light, presenting superior activity in comparison with the TiO_2_-1% Fe–N, pH ~5.5 sample. An exponential degradation of MB can be observed for both photocatalysts; moreover, no saturation tendency existed in visible light during 150 min of measurement. The P25-based samples exhibit much lower activity in both UV and visible light regions (red and green curves in Figure 6).

#### 2.1.6. Hydrodynamic Size and Colloidal Stability Behavior

A complete characterization of NPs suspensions is essential before the biological tests in order to obtain valuable information regarding the polydispersity and stability behavior in various media. In this way, the intensity-weighted hydrodynamic average diameter (*Z*-average), polydispersity index (PdI), and zeta potential were determined in the case of fresh NP dispersions and compared with the values obtained after their autoclaving at 120 °C. The results shown in Table 4 revealed the aggregation tendency as shown by SEM images (Figure 4) and also the particles negative charge. The mean size of P25 particles suspended in ultrapure water was above 700 nm which suggested the formation of large aggregates that could not easily be broken by sonication, as it was previously described [34,35,36,37]. In accordance with these findings, the fresh co-doped TiO_2_ NPs dispersions in water showed average sizes above 1000 nm and increased PdI values, which may be due to the doping process. In addition, the zeta potential values reflected the poor stability of these particles.

The characterization performed after autoclaving the suspension in PBS (Table 4) showed almost similar results with the ones of fresh dispersions, suggesting that the process of sterilization at 120 °C and the presence of salts did not induce changes on the aggregation and stability profile. Regarding the NPs behavior in cell culture medium there were important changes noticed which were in accordance with previous works on TiO_2_ NPs [38,39]. A significant increase in average size above 3300 nm was correlated with a PdI value of 1, confirming the poorer dispersion and severe aggregation, possibly due to the high ionic strength of the culture medium, as it was previously discussed [38,39].

Taking into account the very high values of hydrodynamic size and PdI of autoclaved NPs dispersions in cell culture medium (Table 4), we considered that zeta potential could reveal more suggestive information and a more realistic overview regarding the suspensions’ stability and behavior over time. Thus, the measurements obtained at 0, 24, and 72 h, the time end-points used for biological tests, were presented in Table 5. These results demonstrated that the initial relative stability of co-doped TiO_2_ NPs suspensions just before the cell culture exposure, the zeta potential being near the limit of stability of −30 mV, was diminished after the first 24 h. The zeta potential values were further maintained until the end of the incubation time, 72 h. It must be noticed that there was no significant difference between the two types of TiO_2_ NPs suspensions of pH ~5.5 or pH ~8.5.

### 2.2. Antimicrobial Activity

The measurement of the microbicidal activity of the analyzed samples was performed comparatively after 30 min of exposure to ultraviolet and visible light inside the laminar flow cabinets. The samples exposed to visible light showed an improved antimicrobial activity than those exposed to UV, as revealed by the obtained minimal inhibitory concentration (MIC) values (Table 6).

The antimicrobial activity of the powders against the Gram-positive bacterial strains *S. aureus* and *E. faecalis* and the Gram-negative *E. coli* strain revealed the same efficiency with an MIC value of 0.0625 mg·mL^−1^. Regarding *P. aeruginosa*, the powders of TiO_2_-1% Fe–N obtained at different pH values exhibited a high antimicrobial efficiency with very low MIC values of 0.002 mg·mL^−1^.

These two powders have been also very effective against *C. albicans* strain, with MIC values of 0.015 and 0.004 mg·mL^−1^.

In case of the antibiofilm activity of the TiO_2_-1% Fe–N, pH 8.5 sample, the exposure to UV light induced an improvement of the anti-biofilm activity against the tested microbial strains compared to VIS light, excepting *E. coli*, and for the TiO_2_-1% Fe–N, pH 5.5 NPs only against two strains, i.e., *S. aureus* and *C. albicans*. In these cases, the exposure to UV light led to a decrease of the minimal biofilm eradication concentration (MBEC) values by 2–8 times (Table 7).

### 2.3. Effects of TiO_2_ NPs on Cell Viability

**Figure 7:**
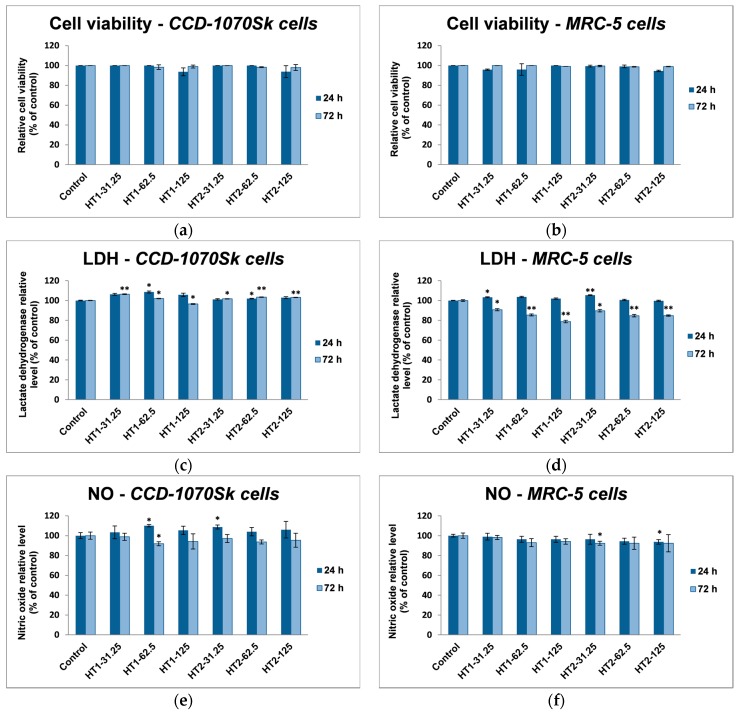
Biocompatibility of different concentrations (μg/mL) of the two TiO_2_ NPs samples: TiO_2_-1% Fe–N NPs co-precipitated at pH 8.5 (HT1) or pH 5.5 (HT2), as shown by cell viability, lactate dehydrogenase (LDH), and nitric oxide (NO) release assays after 24 and 72 h exposure on normal skin (**a**,**c**,**e**) and lung (**b**,**d**,**f**) fibroblasts. Results are expressed as the mean ± standard deviation (SD) (*n* = 3) and represented relative to the untreated cells (control). * *p* < 0.05 and ** *p* < 0.01 compared to control.

The influence of TiO_2_ NPs on the viability and membrane integrity of cells, and the potential of these particles to initiate inflammation were established by in vitro methods assessed on normal human skin and lung fibroblasts. Two intervals of exposure (24 and 72 h) and three different concentrations (31.25, 62.5, and 125 μg/mL) were selected for each of the two photocatalytic TiO_2_-1% Fe–N samples. These concentrations were selected to establish a better correlation between the antimicrobial activity and the potential cytotoxicity on human cells. The toxic effects of TiO_2_ exposure on cell viability were first studied by Trypan Blue assay. The average values of cell number for control after 24 and 72 h were 10.88 ± 0.53 and 21.67 ± 2.36, respectively. These results are expressed as the mean ± standard deviation (SD) of three independent experiments. The cell numbers for the rest of the experimental groups were expressed as percentages from the control values. Three count repetitions were done on the same batch of cells. The statistical analysis was performed using comparisons between groups evaluated by Student’s *t*-test and we obtain values of *p* higher than 0.05, which are not considered statistically significant. Figure 7a,b revealed that, regardless of the time exposure or photocatalyst concentration, both lung and dermal cells showed no significant changes in terms of cell viability.

To better describe the cytotoxicity of TiO_2_ NPs, the lactate dehydrogenase (LDH) leakage level was measured. In the case of dermal fibroblasts, the 24 h exposure to the HT1 sample resulted in an increase of only 5%–8% in LDH release, but no changes for HT2 were observed (Figure 7c). After 72 h of incubation all values were very close to control, proving that TiO_2_-1% Fe–N NPs did not affect the cell membrane integrity. On the other hand, the lung cells showed no significant changes until 72 h of exposure when a slight decrease of LDH level was observed (Figure 7d), which could suggest a proportional decline in cell number compared to the control.

Additionally, the amount of released nitric oxide (NO) was determined. Major differences were not seen between the two cell lines or the two TiO_2_ NPs samples. Thus, the level of NO released by CCD-1070Sk dermal cells in the culture medium registered a slightly increase up to 10% only after 24 h of incubation with both types of TiO_2_ NPs (Figure 7e), while MRC-5 lung cells maintained the amount of NO around the control values no matter of concentration or time exposure (Figure 7f). Therefore, the inflammation induced following the exposure to the tested NPs was too low and cannot be considered significant.

### 2.4. Cellular Antioxidant Defense

The evaluation of antioxidant defense system which is responsible for the cell protection against oxidative damage was established by measuring the activities of the reduced glutathione (GSH) level, and the catalase (CAT), glutathione *S*-transferase (GST), and glutathione peroxidase (GPx).

**Figure 8:**
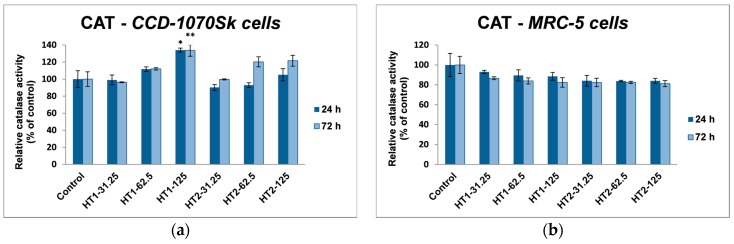
Relative levels of catalase specific activity in normal skin (**a**) and lung (**b**) fibroblasts exposed to different concentrations (31.25, 62.5 and 125 μg/mL) of TiO_2_-1% Fe–N NPs co-precipitated at pH 8.5 (HT1) or 5.5 (HT2) for 24 and 72 h. Results are expressed as the mean ± standard deviation (SD) (*n* = 3) and represented relative to the untreated cells (control). * *p* < 0.05 and ** *p* < 0.01 compared to control.

As it can be seen in Figure 8, there is a significant difference between the two cell lines in catalase activity. Thereby, in dermal fibroblasts the level of CAT activity increased by almost 35% of control after the incubation with 125 µg/mL HT1 for both time intervals (24 and 72 h), while the HT2 sample induced an increase in CAT activity by only 20% after 72 h exposure, which suggested the activation of antioxidant defense mechanism after a longer time exposure. In contrast, TiO_2_-1% Fe–N NPs had a more severe influence on MRC-5 lung cells. Even after the first 24 h of exposure the CAT activity decreased, the decline reaching almost 20% after 72 h.

**Figure 9:**
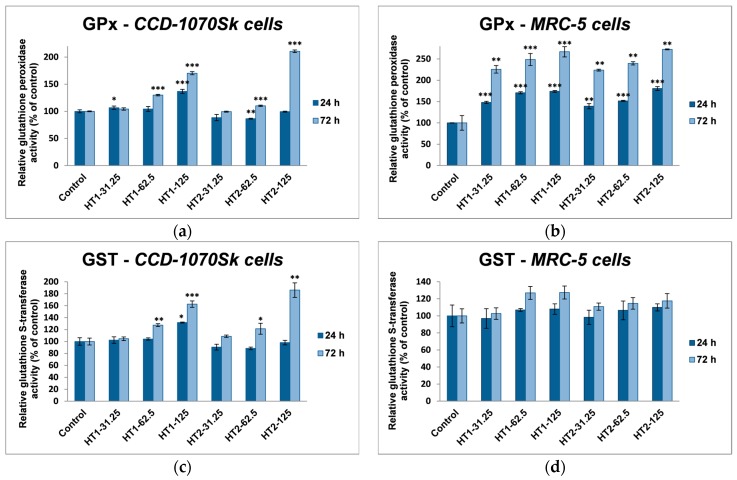

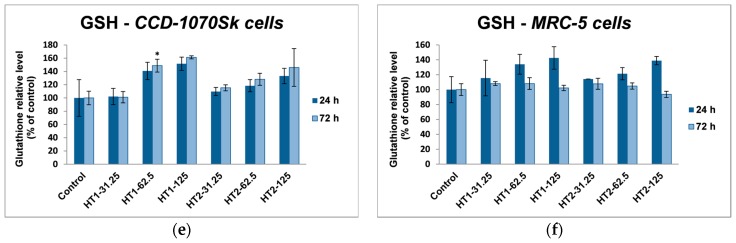
Glutathione-dependent enzymes’ activities and glutathione level in normal skin (**a**,**c**,**e**) and lung (**b**,**d**,**f**) fibroblasts exposed to different concentrations (31.25, 62.5 and 125 μg/mL) of TiO_2_-1% Fe–N NPs co-precipitated at pH 8.5 (HT1) or 5.5 (HT2) for 24 and 72 h. Results are expressed as the mean ± standard deviation (SD) (*n* = 3) and represented relative to the untreated cells (control). * *p* < 0.05, ** *p* < 0.01 and *** *p* < 0.001 compared to control.

Although a significant increase of GPx activity was observed only for the high dose of both types of TiO_2_ NPs in the dermal fibroblasts (Figure 9a), a strong elevated activity was measured for all of the tested concentrations compared to the control in lung cells (Figure 9b). Taking into account that, in dermal fibroblasts, the GST activity profile (Figure 9c) resembles with the GPx one (increased levels for the high dose) being accompanied by an enhancement of GSH content (Figure 9e), it could be suggested the activation of both enzymatic and non-enzymatic antioxidant mechanisms cooperate for a proper cellular defense against oxidative injuries. In contrast, the level of GST activity did not significantly change (about 20% compared to control) after lung cells’ exposure to TiO_2_-1% Fe–N NPs for 24 and 72 h (Figure 9d). These results correlated with a GSH content diminution between the two time intervals (Figure 9f) and the high GPx activity at 72 h underlined that the detoxification process was realized more intense for this type of cells and particularly throughout this enzyme of the antioxidant defense system.

### 2.5. Lipid Peroxidation

**Figure 10:**
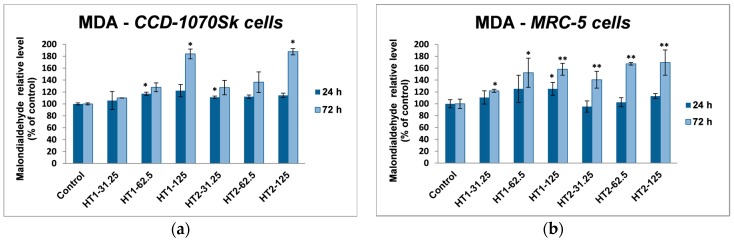
Malondialdehyde (MDA) levels in normal skin (**a**); and lung (**b**) fibroblasts exposed to different concentrations (31.25, 62.5, and 125 μg/mL) of TiO_2_-1% Fe–N NPs co-precipitated at pH 8.5 (HT1) or 5.5 (HT2) for 24 and 72 h. Results are expressed as the mean ± standard deviation (SD) (*n* = 3) and represented relative to the untreated cells (control). * *p* < 0.05 and ** *p* < 0.01 compared to control.

The effects of TiO_2_-1% Fe–N NPs on the lipid peroxidation in human dermal and lung fibroblasts are shown in Figure 10. No major differences were noticed between HT1 and HT2 samples. The malondialdehyde (MDA) content of both cell lines was not significantly changed compared to control after 24 h exposure; the TiO_2_ NPs determined only a dose-dependent slightly increase. In CCD-1070Sk cells, the incubation of 72 h with the nano-photocatalysts led to a MDA level increased by almost 90% for the 125 μg/mL concentration. The lipid peroxidation level in MRC-5 cells after 72 h was a bit lower. However, compared to the skin cells, the lung fibroblasts registered the same increase by 70% of the MDA content for 62.5 and 125 μg/mL doses.

### 2.6. Dynamic Changes of the Actin Cytoskeleton

**Figure 11:**
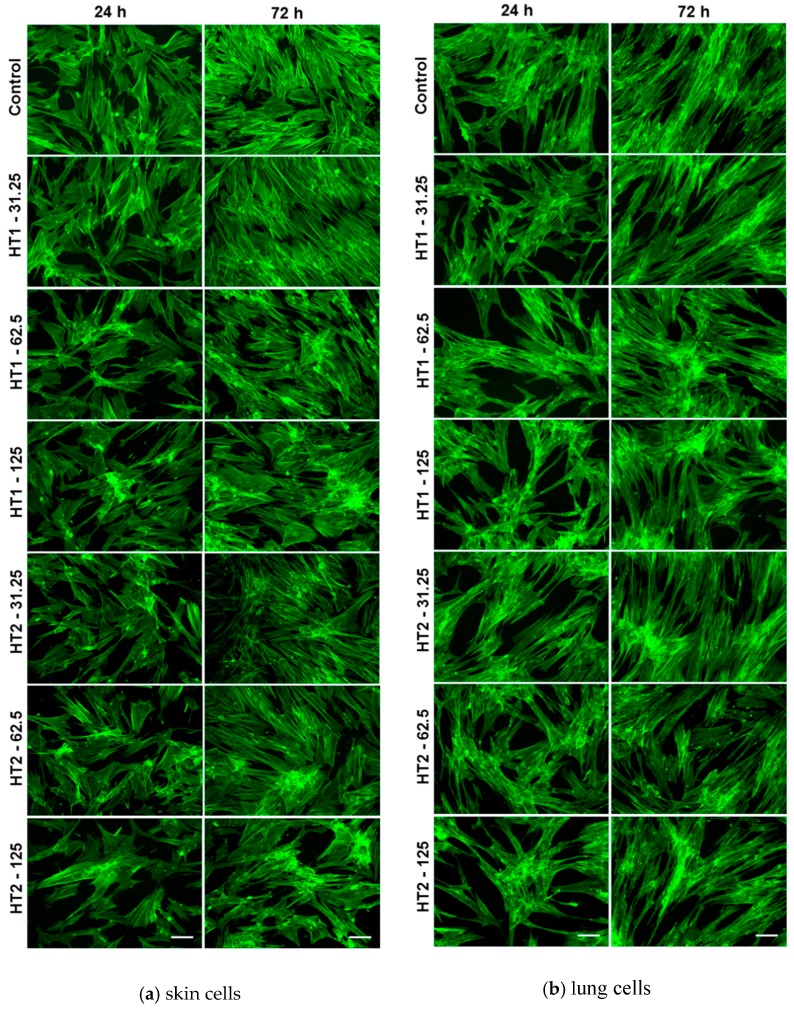
Actin cytoskeleton organization of skin (**a**); and lung (**b**) fibroblasts after 24 and 72 h of incubation with different concentrations (μg/mL) of the two TiO_2_ NPs samples: TiO_2_-1% Fe–N co-precipitated at pH 8.5 (HT1) or pH 5.5 (HT2). F-actin (green) was labeled with phalloidin-fluorescein isothiocyanate (FITC). Scale bar: 100 μm.

The changes in MRC-5 and CCD-1070Sk cell morphology after the exposure to TiO_2_-1% Fe–N NPs were investigated by phase contrast microscopy. The dynamic changes in actin cytoskeleton organization displayed in Figure 11 were in agreement with the findings on cell viability assays revealed in Figure 7. Therefore, the human skin and lung fibroblasts maintained their fibroblast-specific elongated morphology after 24 and 72 h exposure with no significant differences between the samples compared to the control. The multitude of stress fibers proved that human cells established numerous focal adhesions, and their behavior was not notably changed in response to nano-photocatalysts.

## 3. Discussion

The present study describes the synthesis, characterization, and biological evaluation of TiO_2_ nanoparticles co-doped with N–Fe for an increase of photoactivity in the visible light. The synergistic effect of the N–Fe has been previously explained by the fact that nitrogen causes a band-gap narrowing which allows the absorption of longer wavelengths from visible light region, whereas iron increases the efficiency of charge separation [40].

Due to its high photoreactivity, TiO_2_ has been widely used as a photocatalytic agent, to kill various groups of microorganisms, including bacteria, fungi, and viruses [41]. The antibacterial effect occurs as an outcome of the ROS interaction with bacterial structural components. ROS especially attack the unsaturated phospholipids from the the cell membrane, lipid peroxidation being considered the lethal mechanism of the photocatalytic process [42]. What makes photocatalytic sterilization the most effective technique compared to conventional antibacterial treatments is the complete mineralization to CO_2_ and H_2_O of dead bacteria and the toxins released by them [43].

There are also scarce reports showing that doping of TiO_2_ NPs with N, Fe, or both induces an improvement of their antimicrobial properties. Thus, N-doped TiO_2_ exhibited improved antibacterial properties against *E. coli* and *B. subtilis* under fluorescent light irradiation [44]. N–Fe co-doped TiO_2_ in the form of powders and nanofilms proved to exhibit a good bactericidal activity against *E. coli* under visible light irradiation [45,46].

The purpose of our study was to obtain biocompatible TiO_2_-based nanoparticles with increased photocatalytic efficiency. The morphological, catalytic, and optical properties of particles depend on their shapes and sizes which are mainly established by the physico-chemical parameters used within the synthesis method. Herein, the pH values during the synthesis obviously change, as expected, with the relative phase content (anatase/brookite), the crystallite and the particle size, as well as the photocatalytic behavior of the powders, as shown by XRD, TEM, and photocatalytic analyses (Figure 1, Figure 3 and Figure 6). Thus, only the hydrothermally-synthesized TiO_2_-1% Fe–N NPs (pH 8.5 and 5.5) were selected herein for the biological assessments because the P25 commercial product and P25-based powders did not show significant photocatalytic activity (Figure 6) and were also previously analyzed in other studies [47,48,49,50,51,52]. Before the biological tests, the complete characterization of co-doped TiO_2_ NPs suspensions in cell culture medium revealed their polydispersity and the decrease in stability over time. The surface of TiO_2_ nanoparticles dispersed in water is covered by hydroxyl group [53] and, the ions present in solution are adsorbed on the particle, changing the surface charge and the electric potential between the particle surface and the dispersion solvent [54].

We investigated the antimicrobial activity of the nano-photocatalysts synthesized in our work on a wide range of microbial strains including bacteria and yeasts, in planktonic and adherent states. The bactericidal activity of the obtained powders was improved under exposure to visible light compared to UV, the highest promising results sustained by the very low minimal inhibitory concentration (MIC) values being obtained against the Gram-negative *P. aeruginosa* bacterial strain and *C. albicans* yeast strain. To our best knowledge, the anti-biofilm activity of N–Fe co-doped TiO_2_ NPs is reported for the first time in this paper. Similar to the activity against planktonic bacteria, the exposure to either visible or UV light improved the efficiency of the photocatalytic NPs against the microbial strains grown in biofilm. The anti-biofilm activity was preserved in many cases at sub-inhibitory concentrations. The MBEC values were much lower compared to MIC values, suggesting the possible usage of these compounds as anti-pathogenic agents, i.e., compounds which do not interfere with the microbial growth, but are able to inhibit the microbial adherence and biofilm development. By this mechanism of action, they could prevent the negative consequences of biofilm formation, paving the way for a large spectrum of applications for the water supply systems, food, cosmetic, and pharmaceutical industries, as well as in different medical fields.

Taking into consideration the enlarged interest for photocatalytic applications of TiO_2_ NPs in various fields, it is essential to investigate their biological response initiated after cell exposure. Lately, a number of studies performed on different cell lines revealed that small size TiO_2_ NPs (10–20 nm) are more harmful and reactive, even without photoactivation, while normal-sized TiO_2_ is biologically inert [55,56]. Additionally, the adverse effects of TiO_2_ NPs have been investigated in detail at both in vitro and in vivo scales. It was reported that TiO_2_ NPs cause cytotoxicity in human bronchial epithelial cells [22], human lung fibroblasts [57], and brain microglia [49]. TiO_2_ NPs exposure on aquatic species caused oxidative damage-mediated effects in *Cyprinus carpio* [58], *Oncorhynchus mykiss* [59] and *Daphnia magna* [60]. Thus, our study aimed to fill the current knowledge by investigating the cytotoxic effects induced by new;y-developed TiO_2_ NPs in MRC-5 and CCD-1070Sk cells.

Currently, there are several paradigms regarding NPs-mediated toxicity, which include cell death, inflammation, inhibition of cellular proliferation, or DNA damages, but among these the most discussed is oxidative stress [61]. It occurs as a result of the disproportion between the rate of ROS molecules formation and the cellular antioxidant capacity to neutralize them [62]. Due to their physicochemical properties, TiO_2_ nanoparticles are able to deposit on the cell surface or in cellular organelles where they interact with the structural components [63].

The cells can maintain the ROS level under normal metabolic conditions and prevent oxidative stress by activating enzymatic and non-enzymatic antioxidant systems [64]. The first line of defense is provided by superoxide dismutase and catalase, which convert ROS into less damaging compounds, followed by glutathione peroxidase and glutathione S-transferase, which detoxify the intermediate products [65]. However, only the up-regulation of these antioxidant enzymes is not sufficient to neutralize the ROS generation by TiO_2_ NPs in dermal and lung fibroblasts. Glutathione is a tripeptide which acts like a free-radical scavenger and has a key role in these intracellular antioxidant defense processes [66]. Due to its active thiol group GSH can directly interact with ROS and it acts as a cofactor for GPx and GST enzymes. Moreover, because of its importance in cell defense, GSH is also a vulnerable target in front of different harmful agents, which can directly deplete or inactivate it [67].

In our experiments, TiO_2_-1% Fe–N NPs increased the specific activities of antioxidant enzymes in both cell lines, which proved that they induced oxidative stress and the lung and dermal fibroblasts responded by activating their antioxidant defense system. The CAT activity level decreased only in MRC-5 lung fibroblasts. Additionally, Turkez et al. suggested that a decline of CAT activity could be correlated with an increased genotoxic effect [68]. Our results could prove that the H_2_O_2_ amount generated by oxidative stress was low and, as a result, this was decomposed by GPx, which has a lower *K*_M_ for H_2_O_2_ [69]. GPX and GST activities were significantly elevated in MRC-5 and CCD-1070Sk cells, especially after 72 h, when a slight diminution of the GSH level occurred in MRC-5 cells at the highest concentrations of both TiO_2_-1% Fe–N samples. These findings indicated that GPX and GST might have an important influence in counteracting the oxidative stress induced by TiO_2_ NPs. Previous reports showed diminished GSH level in different types of cells after TiO_2_ NPs exposure [70,71]. We noticed an insignificant decrease of GSH levels only in MRC-5 cells incubated with nanoparticles for 72 h, which suggested that photocatalysts were well tolerated. Furthermore, an increase in lipid peroxidation level was noticed in both skin and lung cells (Figure 10). The enhancement of lipids peroxidation by nano TiO_2_ was the result of an oxidative attack which was initiated by the reduction of the cellular antioxidant defense mechanism. Similar results were reported in kidney cells exposed to TiO_2_ NPs [72].

## 4. Materials and Methods

### 4.1. Synthesis and Characterization of Titanium Dioxide Nanoparticles

P25 powder (Sigma-Aldrich, St. Louis, MO, USA) was impregnated with 1% Fe and N atoms as follows: suitable quantities of TiO_2_, FeCl_3_·6H_2_O, and urea were dispersed/dissolved via mechanical and ultrasonic stirring in distilled water. The resulted mix was heated at 200 °C for two hours in a Teflon-lined autoclave. The resulted powder was rinsed with ultrapure water to eliminate salts (until pH ~6.5), dried, and finally calcined for 2 h at 400 °C.

For the synthesis of TiO_2_-1% Fe–N particles at pH 5.5 and 8.5 suitable amounts of TiCl_3_ and FeCl_3_·6H_2_O were mixed using energetic stirring in ultrapure water; the pH values were adjusted with 25% NH_4_OH solution. Then, the obtained Ti(III) precipitates were oxidized in air until the color changed from violet to white. To remove salts, the Ti(IV) and Fe(III) hydroxides that co-precipitated were washed with ultrapure water and dried in air at 105 °C. The nitrogen doping was performed by the hydrothermal treatment (200 °C/2 h) of the resulted co-precipitate in the presence of urea. The dried resulted powder was calcined at 400 °C for 2 h in air.

XPS testing was performed in an analysis chamber equipped with a 150 mm hemispherical electron energy analyzer (Phoibos, SPECS Gmbh, Berlin, Germany), a dual anode (Mg/Al Kα) X-ray source, and a monochromatized (Al Kα/Ag Lα) X-ray source, part of a complex surface science cluster (Specs). Monochromatized Al Kα1 (1486.74 eV) radiation was used to extract the electrons from the sample. The analyzer operated in fixed transmission mode with pass energy of 20 eV; the estimated combined (source + analyzer) resolution was about 0.75 ± 0.025 eV. Sample neutralization was achieved using an electron flood gun operating at 1 eV energy and 100 μA electron current. The energy scale was calibrated to the “standard” value of C 1s (284.6 eV) acknowledged by the XPS community. The base pressure during the measurements was about 10^−9^ Pa.

TEM investigations were performed using a transmission electron microscope (JEOL JEM ARM200F, Tokyo, Japan) operated at 200 kV, on samples obtained by crushing the powders in ethanol, dispersing them by sonication and dropping on lacey carbon grids.

The surface morphology of tested particles was investigated with a scanning electron microscope (SEM; Quanta 200, FEI, Eindhoven, The Netherlands) with a large field detector (LFD) operating at 15 kV. The particles were suspended in absolute ethylic alcohol and sonicated for one hour. A drop of suspension was spread on a thin glass plate and dried under a UV lamp.

The diffuse reflectance spectra were recorded on Jasco V550 UV-VIS spectrophotometer (Jasco, Inc., Tokyo, Japan), using nanoparticles powder and BaSO_4_ as a reflectance sample.

In order to study the photocatalytic behavior of co-doped titania nanoscaled powders, we prepared three layered films as follows: The nanopowders were dispersed in distilled water and sonicated in an ultrasonic bath. The final concentration of photocatalytic powder in water was 1 mg/mL. Then, a suitable amount of suspension (calibrated drops) were deposited carefully, to cover the quartz buffer in the horizontal position. The film was dried at 30 °C in the absence of any air current. A new photocatalytic layer (the same number of calibrated drops) was deposited after completely drying. The procedure was three times repeated to obtain a three-layer nanoscaled co-doped TiO_2_ film. The resulted films were dried in air for 16 h and then cleaned with a 30 W UV (385 nm) lamp for 2 h to remove possible organic contamination. Finally, the films were immersed in methylene blue and dried in the dark at room temperature. We have to mention that within this deposition procedure no additional thermal treatment was necessary, therefore, no structural changes could occur during film’s preparation. In order to reveal the activity in both UV (368 nm) and visible (λ > 400 nm) spectral regions, the obtained films were tested using a PCC-2 (ULVAC RIKO, Chigasaki, Kanagawa, Japan) photocatalytic checker.

In order to assess the hydrodynamic size and zeta potential, the TiO_2_-based NPs dispersions were prepared at a concentration of 1 mg/mL in ultrapure water and at a concentration of 125 µg/mL in phosphate saline buffer (PBS) or cell culture media, using 5 min ultrasonication and then were analyzed on a Malvern Zetasizer Nano-ZS instrument (Malvern Instruments, Malvern, Worcestershire, UK) by dynamic light scattering (DLS) and laser Doppler velocimetry (LDV) technologies. Three measurements were taken for each sample to determine the particles’ size and zeta potential.

### 4.2. Antimicrobial Activity Assays

The influence of the obtained powders on the prokaryotic and eukaryotic microbial strains was tested using Gram-negative bacterial strains (*E. coli* ATCC 8739, *P. aeruginosa* ATCC 27853), Gram-positive bacterial strains (*S. aureus* ATCC 6538, *E. faecalis* ATCC 29212), and fungal strains (*C. albicans* ATCC 10231). For this purpose, microbial suspensions with a density of 1.5 × 10^8^ colony forming units (CFU)·mL^−1^ equivalent to the 0.5 McFarland’s turbidimetry standard were obtained starting from fresh culture of 15 to 18 h developed on solid media. The tested powders were suspended in dimethyl sulfoxide (DMSO) in order to obtain the stock solution with a concentration of 10 mg·mL^−1^. The quantitative assay of the antimicrobial activity was performed by liquid medium micro-dilution method in 96-well plates. In this purpose, two-fold serial dilutions of the nanoparticles’ suspensions, with a concentration range from 1000 to 4 μg·mL^−1^ were achieved in a volume of 200 μL of liquid culture medium, irradiated for 30 min in UV (100–290 nm) and VIS light, respectively, using the laminar flow germicidal and illuminating lamps, and then, each well was seeded with 50 μL standardized microbial inoculum. Positive controls (for bacterial culture) were used. After 24 h of incubation at 37 °C, the MIC values were read as the powder concentration found in the last well in which the visible overnight growth of the microbial culture was lower as compared to the positive control, and confirmed by a decreased value of absorbance at 600 nm (Apollo LB 911 ELISA plate reader) [73].

For the anti-biofilm activity evaluation of the tested nanopowders the microtiter plate method was used. In this purpose, after reading the micro-plates for the establishment of the MIC values, their contents were emptied and, then, the plates were washed three times by phosphate-buffered saline. The microbial cells forming a biofilm adhered on the plastic wells wall resisted to washing and were with fixed cold methanol, which was left to act for 5 min then, colored by crystal violet solution for 15 min and finally resuspended in a 33% acetic acid solution. The density of the microbial biofilm harvested from the plastic wells was measured by reading the optical density at 490 nm for the colored suspensions. The minimal biofilm eradication concentration (MBEC) value was corresponding to the concentration found in the well in which the absorbance values were inferior to those of the positive control [74].

### 4.3. Cell Culture

Normal human fibroblasts from skin (CCD-1070Sk cell line, ATCC Cat. No. CRL-2091) and from lung (MRC-5 cell line, ATCC Cat. No. CCL-171) were grown in complete Eagle’s minimum essential medium (MEM; Gibco/Invitrogen, Carlsbad, CA, USA) with the addition of 10% fetal bovine serum (FBS; Gibco/Invitrogen, Carlsbad, CA, USA) at 37 °C in a humidified atmosphere with 5% CO_2_. The culture medium was replaced every two days with a fresh medium until cells reached 80% confluence, when a 0.25% (*w*/*v*) Trypsin 0.53 mM EDTA solution (Sigma-Aldrich) was used to split the cells for future sub-cultivations.

### 4.4. Culture Treatment Protocol

Two stock suspensions of TiO_2_ nanoparticles doped with 1% Fe–N atoms synthesized at pH values of 8.5 and 5.5 were prepared in PBS and sterilized by autoclaving at 120 °C for 20 min. The fibroblasts were counted and cultured at a density of 2 × 10^4^ cells/cm^2^ into 24-well plates (for biocompatibility assessment) or in 75 cm^2^ culture flasks (for antioxidant enzymes assays, glutathione content, and lipid peroxidation analysis) and allowed to adhere overnight. Afterwards, cells were exposed to various concentrations (31.25, 62.5, and 125 µg/mL) of TiO_2_-1% Fe–N nanoparticles for 24 and 72 h. Controls represented by untreated cells were used for each test.

### 4.5. Cell Viability

In order to measure the cell viability, the cells were detached as it was described in Section 4.3 and counted using Trypan Blue staining. Therefore, 15 μL of cell suspension were mixed with an equal volume of 0.4% (*w*/*v*) Trypan Blue solution prepared in 0.81% NaCl and 0.06% (*w*/*v*) dibasic potassium phosphate (Sigma-Aldrich). The cells’ counting was performed using a dual-chamber hemocytometer and a light microscope. Cell viability was given by the following formula:

% viable cells = [1.00 − (Number of blue cells/Number of total cells)] × 100

The cell membrane integrity determined by the LDH amount released in culture medium was assessed using a commercial kit (TOX7, Sigma-Aldrich) according to the manufacturer’s instructions. Thus, 50 μL of culture supernatants were homogenized with 100 μL of mix consisting of equal parts of dye, substrate and cofactor, and incubated for 30 min in dark. The reaction was stopped by adding 15 μL of 1 N HCl, and then the absorbance was read at 490 nm using a GENios Tecan microplate reader (TECAN GENios, Grödig, Austria).

The inflammatory potential determined by the level of NO released in the culture medium was measured using the Griess reagent, which is a stoichiometric solution (*v*/*v*) of 0.1% naphthylethylendiamine dihydrochloride and 1% sulphanilamide in 5% H_3_PO_4_. Therefore, culture supernatants were mixed with an equal volume of Griess reagent and absorbance was read at 550 nm using a GENios Tecan microplate reader. Finally, NO concentration was calculated by extrapolation on a NaNO_2_ standard curve.

Cell spreading and actin cytoskeleton morphology were observed via fluorescence microscopy. Firstly, the cells were fixed with 4% paraformaldehyde for 20 min and permeabilized with 0.1% Triton X-100−2% bovine serum albumin (BSA) for 1 h, following later to mark filamentous actin (F-actin) marked with 20 μg/mL phalloidin conjugated with FITC (Sigma-Aldrich, Munich, Germany). Images were obtained with an inverted fluorescence microscope Olympus IX71 (Olympus, Tokyo, Japan).

### 4.6. Preparation of Cell Lysates

MRC-5 and CCD-1070Sk cells were collected from culture flasks, washed with PBS and the cell lysates were obtained by sonication (30 s × 3 times) on ice with an ultrasonic processor (Hielscher UP50H, Teltow, Germany). The homogenate was centrifuged at 3000× *g* for 10 min at 4 °C and the supernatants (total proteic extracts) were collected for biochemical assays.

### 4.7. Protein Concentration Assay

The protein concentration of the cellular extract was measured according to the method described by Bradford [75] using the Bradford Reagent (Sigma-Aldrich) and a BSA standard curve.

### 4.8. Antioxidant Enzymes Assays

The activities of antioxidant enzymes were determined by spectrophotometric methods. Catalase (CAT) (EC 1.11.1.6) activity was measured by monitoring the decrease in absorbance of H_2_O_2_ at 240 nm, as described in Aebi’s method [76]. One unit of CAT activity represented the amount of enzyme that catalyzed the conversion of 1 μmol of H_2_O_2_ in 1 min under standard conditions. Glutathione peroxidase (GPX) (EC 1.11.1.9) activity was assessed according to the method of Beutler [77] by monitoring at 340 nm a coupled reaction with glutathione reductase that catalyzed NADPH oxidation. One unit of GPx activity was defined as the quantity of enzyme that catalyzes the transformation of 1 μmol of NADPH per minute. Glutathione S-transferase (GST) (EC 2.5.1.18) activity was determined as described in the method of Habig et al. [78] by measuring the rate of 1-chloro-2,4-dinitrobenzene (CDNB) conjugation with GSH at 340 nm. One unit of GST activity was defined as the amount of enzyme that produced 1 μmol of conjugated product per minute. All data were obtained in physiological conditions at a pH value of 7.4 and room temperature (25 °C) and the final results were calculated as specific enzymatic activities (units/mg of protein) and expressed as a percent of the control levels.

### 4.9. Glutathione Content

The proteins from cell lysates were precipitated with a 5% sulfosalicylic acid solution (Sigma-Aldrich) (1:1) and removed by centrifugation at 10,000 rpm and 4 °C for 10 min. The GSH content was determined using the commercial glutathione assay kit (Sigma-Aldrich) according to the protocol provided by the manufacturer. Briefly, each sample was incubated with 5,5′-dithiobis-2-nitrobenzoic acid (DTNB) for 5 min at room temperature in order to allow the reduction of DTNB into 5-thio-2-nitrobenzoic acid (TNB). The absorbance was recorded at 405 nm using a microplate reader (TECAN GENios, Grödig, Austria). The GSH levels were calculated as nmols/mg protein and the results were expressed relative to control.

### 4.10. Lipid Peroxidation

Lipid peroxidation expressed by malondialdehyde (MDA) level was assessed using the fluorimetric method described by Dinischiotu et al. [79]. A volume of 200 µL of cell lysate diluted correspondingly was mixed with 700 µL of 0.1 N HCl and incubated for 20 min at room temperature. Forwards, 900 µL of 0.025 M thiobarbituric acid (TBA) was added and the mixture was incubated for 65 min at 37 °C. Subsequently, relative fluorescence units (RFU) recorded (excitation wavelength = 520 nm; emission wavelength = 549 nm) (FP-750 Spectrofluorometer, Jasco, Tokyo, Japan) were converted to nmols malondialdehyde (MDA) using a 1,1,3,3-tetramethoxypropane standard curve. The MDA concentration was expressed as nmols of MDA/mg protein and all the results were represented relative to control.

### 4.11. Statistical Analysis

All results were represented as mean value ± standard deviation (SD) of three different experiments. The statistical analysis was performed using comparisons between groups evaluated by Student’s *t*-test or two-way ANOVA, followed by Bonferroni post hoc test using GraphPad Prism software (version 5; GraphPad Software, Inc., La Jolla, CA, USA), and only a value of *p* less than 0.05 was considered statistically significant.

## 5. Conclusions

Herein, we succeeded to obtain co-doped TiO_2_ nanoparticles with increased photocatalytic efficiency. By modifying the pH values during the synthesis, the relative phase content (anatase/brookite), the crystallite and the particle size, as well as the photocatalytic behavior of the powders, were obviously changed. Our results demonstrated the biocompatibility of TiO_2_-1% Fe–N nanoparticles at low doses on lung and dermal cells, which may initiate oxidative stress in a dose-dependent manner, providing useful information for future photocatalytic applications. In addition, these particles exhibited significant microbicidal and anti-biofilm activity, suggesting their potential application for microbial decontamination of different environments from the industrial, food, pharmaceutical, and medical fields.

## Figures and Tables

**Table 1:** Lattice parameters, crystallite size, phase assignment, and relative abundance of the P25 and hydrothermally-synthesized samples.

Sample	Lattice Parameters (Å)			Crystallite Size (nm)	Phase Assignment/Abundance (*wt* %)
	*a*	*b*	*c*		
P25	3.7891	-	9.5165	32.8	Anatase (83.1)
	4.5977	-	2.9598	71.0	Rutile (16.9)
P25-1% Fe–N	3.7876	-	9.5139	28.6	Anatase (83.0)
	4.5942	-	2.9632	36.4	Rutile (17.0)
TiO_2_-1% Fe–N (pH ~5.5)	3.7907	-	9.4743	10.4	Anatase (79.4)
	9.1629	5.4437	5.1809	11.6	Brookite (20.6)
TiO_2_-1% Fe–N (pH ~8.5)	3.7912	-	9.4909	12.3	Anatase (85.3)
	9.1429	5.4215	5.2450	8.5	Brookite (14.7)
Errors	±0.0005	±0.0005	±0.0005	±1.5	±1.4

**Table 2:** The binding energy values extracted from the deconvolutions of the X-ray photoelectron spectroscopy (XPS) spectra.

Sample	Ti 2p_3/2_	Fe 2p_3/2_	N 1s
	Binding Energy (eV)		
P25	458.65	-	-
	459.53		
P25-1% Fe–N	458.08	710.40	399.62
	459.30		401.19
	460.27		
TiO_2_-1% Fe–N (pH ~8.5)	456.88	710.55	398.60
	458.62		400.50

**Table 3:** The particle average size of the P25 and hydrothermally-synthesized samples. The results are expressed as mean ± standard deviation.

Sample	Particle Size (nm)
P25	29 ± 15
P25-1% Fe–N	30 ± 10
TiO_2_-1% Fe–N (pH ~5.5)	15 ± 2.8
TiO_2_-1% Fe–N (pH ~8.5)	10 ± 4

**Table 4:** The *Z*-average, polydispersity index (PdI) and zeta potential values measured for fresh and autoclaved particle dispersions.

Sample		Size (d.nm)	Pdl	Zeta Potential (mV)
Fresh dispersions in ultrapure water	P25	765	0.599	−41.0 ± 0.00
	TiO_2_-1% Fe–N pH~5.5	1168	0.783	−12.4 ± 2.83
	TiO_2_-1% Fe–N pH~8.5	1692	0.686	−17.9 ± 1.34
Autoclaved dispersions in PBS	TiO_2_-1% Fe–N pH~5.5	1163	0.598	−12.8 ± 0.92
	TiO_2_-1% Fe–N pH~8.5	1698	0.517	−17.3 ± 1.56
Autoclaved dispersions in cell culture medium	TiO_2_-1% Fe–N pH~5.5	3309	1.000	−28.0 ± 0.64
	TiO_2_-1% Fe–N pH~8.5	3787	1.000	−29.2 ± 3.96

**Table 5:** The zeta potential values measured over time for particle dispersions prepared in cell culture media.

Sample	Zeta Potential (mV)–0 h	Zeta Potential (mV)–24 h	Zeta Potential (mV)–72 h
TiO_2_-1% Fe–N pH ~ 5.5	−28.0 ± 0.64	−8.7 ± 0.92	−8.94 ± 1.17
TiO_2_-1% Fe–N pH ~ 8.5	−29.2 ± 3.96	−8.1 ± 1.53	−7.86 ± 0.29

**Table 6:** The MIC (mg·mL^−1^) values of the TiO_2_-1% Fe–N nanoparticles against the tested microbial strains.

Samples	*S. aureus*		*E. faecalis*		*E. coli*		*P. aeruginosa*		*C. albicans*	
	VIS	UV	VIS	UV	VIS	UV	VIS	UV	VIS	UV
TiO_2_-1% Fe–N, pH ~8.5	0.0625	>1	0.0625	>1	0.0625	>1	0.002	>1	0.015625	>1
TiO_2_-1% Fe–N, pH ~5.5	0.0625	>1	0.0625	>1	0.0625	>1	0.002	>1	0.004	>1

**Table 7:** The MBEC (mg·mL^−1^) values of the TiO_2_-1% Fe–N nanoparticles against the tested microbial strains.

Samples	*S. aureus*		*E. faecalis*		*E. coli*		*P. aeruginosa*		*C. albicans*	
	VIS	UV	VIS	UV	VIS	UV	VIS	UV	VIS	UV
TiO_2_-1% Fe–N, pH ~8.5	>1	0.032	>1	0.5	0.002	0.5	>1	0.25	>1	0.125
TiO_2_-1% Fe–N, pH ~5.5	>1	0.008	>1	1	>1	>1	>1	1	1	0.25
